# Acceptance Rates and Beliefs toward COVID-19 Vaccination among the General Population of Pakistan: A Cross-Sectional Survey

**DOI:** 10.4269/ajtmh.21-0297

**Published:** 2021-09-03

**Authors:** Farah Yasmin, Waleed Asghar, Maryam Salma Babar, Hiba Khan, Shoaib Ahmad, Zaid Hameed, Muhammad Sohaib Asghar, Hamza Nauman, Abdullah Khan Khattak, Zara Arshad, Syed Muhammad Ismail Shah, Sarush Ahmed Siddiqui, Muhammad Osama, Muhammad Samsoor Zarak

**Affiliations:** ^1^ Department of Internal Medicine, Dow Medical College, Dow University of Health Sciences, Karachi, Pakistan;; ^2^ City Gynae Hospital Toba Tek Singh, Medicine, Toba Tek Singh, Punjab, Pakistan;; ^3^ Dubai Medical College for Girls, Faculty of Medicine, Dubai, United Arab Emirates;; ^4^ General Medicine and Surgery, Punjab Medical College, Faisalabad, Pakistan;; ^5^ Department of Internal Medicine, Allied Hospital, Faisalabad, Pakistan;; ^6^ Department of Internal Medicine, Dow University Ojha Hospital, Karachi, Pakistan;; ^7^ Department of Internal Medicine, Aga Khan University, Karachi, Pakistan;; ^8^ Department of Internal Medicine, Capital Hospital, Islamabad, Pakistan;; ^9^ Department of Internal Medicine, Ziauddin Medical University, Karachi, Pakistan;; ^10^ Department of General Surgery, Dow University of Health Sciences, Karachi, Pakistan;; ^11^ World Health Organization, Zhob, Pakistan

## Abstract

Developing countries like Pakistan have previously suffered from barriers to acceptance of vaccination by the public because of financial and belief barriers. This study aims to explore these beliefs and highlight concerns regarding vaccine hesitancy in the general population of Pakistan since they are a hindrance to an effective coronavirus disease-19 (COVID-19) immunization in the country. A cross-sectional study was performed involving 1,778 participants from all four provinces of Pakistan. Results from the study showed more than half of the participants to be unsure of the safety (50%) and efficacy (51%) of the vaccine, whereas 42% were concerned about the side effects of the vaccine. About 72% of the respondents planned to get vaccinated, whereas 28% refused to do so. Internationally made imported vaccines were more trusted by the participants. Forty-four percent of the participants agreed to receive the vaccine upon recommendation from a physician. Lastly, participants who believed in the efficacy of the polio vaccination also considered the COVID-19 vaccine to be safe and effective.

## INTRODUCTION

Coronavirus disease-2019 (COVID-19), caused by the severe acute respiratory syndrome coronavirus-2 (SARS-CoV-2) is suspected to be have originated in the city of Wuhan, China, and was declared as a pandemic by the WHO on March 11, 2020.^1^ Since its discovery on December 12, 2019, the disease has affected the whole world killing millions of people to this day.^2^ The outbreak has also caused huge negative impacts on the public health system as well as disrupting the perception of public health according to many people.^3^^,^^4^ Currently, there is no specific cure in the treatment of COVID-19 as the effects of different medications are still being investigated in clinical trials.^5^ The current treatment is that of supportive care such as oxygenation and fluid therapy, and preventive methods to reduce the spread of the highly transmissible SARS-CoV-2.^6^

According to the Centers for Disease Control and Prevention (CDC), all authorized vaccines are efficacious against symptomatic, laboratory confirmed (65–95%) COVID-19 infections, and can prevent hospitalization from severe COVID-19 infection (≥ 89%).^7^ The BNT162b2 vaccine in its phase 3 clinical trial showed an efficacy of 90.5% in preventing COVID-19, 2 to 7 days after the second dose.^8^ Similarly, the mRNA-1273 had shown an efficacy of 94.1% in preventing symptomatic disease, with a median follow-up of 2 months after completion of the two-dose regimen.^9^ However, there results only show short-term efficacies of the vaccines. Since there is no clinical data on the long-term efficacy of the COVID-19 vaccines, it can perhaps be predicted using the decay in neutralizing titers following vaccination. One study found a nonlinear strong relationship between the mean in-vitro neutralization levels, and the reported efficacy of seven vaccines from their phase 3 clinical trials, namely mRNA-1273, NVX-CoV2373, BNT162b2, rAd26-S+rAd5-S, ChAdOx1 nCoV-19, Ad26.COV2. S, and CoronaVac.^10^

This model compared the decay in the neutralization titers with the convalescent or vaccination cohorts by fitting a model of exponential decay. A similar decay in neutralization titers was found induced by vaccination, and from a natural infection by SARS-CoV-2. Therefore, they estimated the half-life of decay in neutralization titers using a study on convalescent subjects, and their data of up to 8 months after infection. They found an estimated half-life of 1,08  days of neutralizing antibodies after vaccination, which was then used to model a 250-day protection, and decay in neutralization after vaccination. Their model predicted that a high initial starting efficacy of 90% of a vaccine would drop to 70% by 250 days, while a starting efficacy of 70% would drop to 33% after 250 days.^10^ These findings suggest that immune responses might wane over time. Their model also gave a prediction of the long-term efficacy of the vaccines. Since they demonstrated their model by suggesting that a natural antibody response is similar to the one induced by vaccination, it can be supported by the fact that the BNT162b2 vaccine was shown to have a lower efficacy against the B.1.351 variants of SARS-CoV-2. Much lower titers of neutralizing antibodies were developed in only 60% and 70% of people after week 1 and week 3 of the second dose of the vaccine, respectively, suggesting a loss of potency of the vaccine-induced neutralizing titers because of the E484K mutation in the variant.^11^ Furthermore, the neutralization level needed to prevent severe disease is fortunately six times lower than that needed to have protection from any symptomatic disease,^10^ meaning that vaccination would still provide protection against severe disease even at much lower antibody levels. A booster might still become necessary after 8–12 months, as suggested by the Pfizer.^11^ Randomized control trials can also overestimate the protection provided by vaccines compared with the real-world where logistical problems exist, such as nonoptimal temperature storage of vaccines, and delays in administering the second dose.^12^

It would not be economically feasible to vaccinate entire populations at one time for every couple of months. More so, it is clear that humans cannot go with social distancing, and other preventive measures for a long period of time either. Hence, the only strategy to manage this ongoing pandemic is to develop a COVID-19 vaccine that can provide long-term clinical benefits along with socioeconomic benefits. A successful COVID-19 vaccine should pass clinical safety, efficacy, large-scale distribution, and production.^13^ Around 15 vaccines are still under trial while many are already under distribution with some participants completing the course of immunization. With many vaccines available, and others still on the rise, there is much dispute about their true efficacy and reliability.

Although great effort is rendered into developing and deploying vaccines, the vaccine coverage rate among the population is also an important factor that decides successful immunization.^14^ Generally, vaccine development would take years with thorough trials for safety measurements before global distribution. Hence, the public acceptance for a multitude of COVID-19 vaccines that were developed within a short period of time remains skeptical. Therefore, vaccine hesitancy is a major hindrance when distributing to certain regions especially the uneducated areas. It has been classified as a major threat to global health by WHO as it disrupts the process of eventually eradicating the disease in question. An approximation of 67% herd immunity may be possible only if vaccination programs are made mandatory coupled with education programs to eliminate the factor of vaccine hesitancy.^15^ However, the recent rise in vaccine hesitancy because of concerns about its safety has led to a slower rise in immunity, and a faster outbreak in many diseases such as measles and polio.^16^ Thus, this conflict affects not only the individual who is hesitant to take the vaccine, but the whole community, making it impossible to reach the goal of successful herd immunization. The overall morbidity and mortality rate of COVID-19 has been high because of rapid virus transmission, and therefore several countries had initiated early research and development for a vaccine. Understanding the specific challenges in carrying out the vaccination across the world will vary from region to region as some may suffer from financial burdens, and others with belief barriers. However, facilitators for vaccine acceptance would help a great deal in improving vaccine coverage rate and are even more vital in a time-dependent pandemic.^17^

Developing countries suffer the most because of inadequate education, and lack of awareness toward public health issues.^18^ The inability to finance vaccination facilities, and vaccine hesitancy makes for a larger struggle to herd immunity.^19^ Pakistan, in particular, has suffered from the failures of previous vaccination programs as a result of fabrications that fed to vaccine hesitancy.^20^ For example, polio is still not eradicated from Pakistan as a result of the misconception of its supposed false side effects because of which the disease now poses a threat to many vulnerable and unprivileged children.^21^ These fabrications turn to stubborn beliefs, and become barriers toward achieving herd immunity among the nation. Furthermore, a combination of other key factors such as severity of the disease, previous vaccination history, lack of belief in healthcare services, route of administration of vaccine, economic and educational status of the individuals, recommendations from doctors, and cost of vaccine also determines the acceptance of vaccines.^22^ In this study, we analyze the acceptance rates of COVID-19 vaccine, beliefs, and barriers that might prevent the participants from being vaccinated against the COVID-19 among the general population in Pakistan. With the previous polio vaccination failure because of the very factor of perceptive barriers, this study aims to prevent the repeat of such drastic public health catastrophes.

## MATERIALS AND METHODS

The cross-sectional study was conducted among the general public of Pakistan between January 28, 2021 and February 11, 2021. All individuals aged ≥ 18 years of either gender, that is, male or female residing in the four provinces of Pakistan i.e., Sindh, Punjab, Balochistan, and Khyber Pakhtunkhwa were included in the survey. All those who refused to provide with an informed consent were excluded. A comprehensive validated self-administered questionnaire was distributed through social media platforms such as WhatsApp, Facebook, and e-mails to the participants. The questionnaire was administered in the native language of the population, that is, Urdu language.

The questionnaire composed of an introductory paragraph stating the primary aim and the objectives of the study, description on voluntary participation, followed by a mandatory informed consent obtained from all participants. The participants were also informed that they could leave the survey at free will and at any time they feel uncomfortable before its completion.

The participants were assured that their anonymity and confidentiality would be maintained as all data provided by the participants would be kept confidential. The outline of the questionnaire was adapted from the previous study conducted by Magadmi and Kamel (2020) with some modifications according to locality.^23^ The questionnaire was reviewed by two senior professors for validity and reliability. The questionnaire was modified based on their recommendations and suggestions. We further carried out a pilot survey on 15 study participants that yielded the questionnaire to be concise, easily understandable, and requiring 3–5 minutes to complete. The questionnaire consisted of five major sections that collected data on sociodemographic parameters, beliefs toward COVID-19 vaccination, COVID-19 vaccine acceptance, potential barriers that may prevent from being vaccinated, and factors that can improve COVID-19 vaccine acceptance. An additional question was created to inquire about the administration of polio vaccine among the individual’s children to compare the beliefs regarding the polio vaccine versus that of the COVID-19 vaccine.

The data was analyzed using the Statistical Package for Social Sciences (SPSS) software package (version 16) (IBM Corporation, Armonk, NY). All categorical variables were reported using frequencies and percentages while mean and standard deviation was used for continuous variables. Univariate logistic regression was applied to access the association of sociodemographic variables, beliefs, barriers, and factors that may improve COVID-19 vaccination with its acceptance rate among the population. A *P* value of ≤ 0.05 was considered as statistically significant.

## RESULTS

### Sociodemographic factors of the participants.

A total of 1,900 questionnaires were distributed to account for incomplete responses out of which 1,778 were completely filled, giving a response rate of 93.6%. The mean age of the participants was 23.29 ± 3.61 years. 625 (35%) were males, 807 (45%) were university graduates, while a minor proportion 156 (9%) was married. Most 473 (27%) earned between 51,000 rupees and 100,000 rupees. The highest proportion of the respondents belonged to Punjab 818 (46%) followed by Sindh 493 (28%). Only 85 (5%) of participants suffered from comorbidities. More than half 979 (55%) self-reported their overall health as good/satisfactory. Upon asking the probability of being infected with COVID-19, majority 824 (46%) stated they do not think they would get the infection in future, while 305 (17%) stated they have been already infected with the virus. The participants were also asked whether they believe in getting their children vaccinated against polio virus to which 419 (24%) showed disapproval (Table 1).

**Table 1:** General characteristics of the participants

General characteristics	Frequency *N* (%)
Age (years) 18–29 30–39 40–60	1,654 (93) 73 (4) 51 (3)
	
Gender Male Female	625 (35) 1,153 (65)
	
Educational status Postgraduate level University graduates Higher secondary Primary/secondary school	107 (6) 807 (45) 763 (43) 101 (6)
	
Occupation Healthcare workers Students Govt/private employees Unemployed	116 (7) 1,458 (82) 132 (7) 72 (4)
	
Marital status Married Single	156 (9) 1,622 (91)
	
Monthly income (rupees) < 20,000 21,000–50,000 51,000–100,000 101,000–200,000 > 200,000	195 (11) 288 (16) 473 (27) 445 (25) 377 (21)
	
Region Sindh Punjab Khyber Pakhtunkhwa Balochistan	493 (28) 818 (46) 348 (20) 119 (7)
	
Any comorbidities Present Absent	85 (5) 1,693 (95)
	
Self-reported overall health Fair Good Excellent	235 (13) 979 (55) 564 (32)
	
What is your best guess as to whether you will get the coronavirus within the next 6 months? I think I will get a severe case of COVID-19 I think I will get a mild case of COVID-19 I don’t think I will get the coronavirus infection I have already had the coronavirus infection	62 (3) 587 (33) 824 (46) 305 (17)
	
Do you believe in getting your children vaccinated against the polio virus? Yes No No response	223 (13) 419 (24) 1,136 (64)

### Acceptance and beliefs toward COVID-19 vaccination.

**Figure 1.**
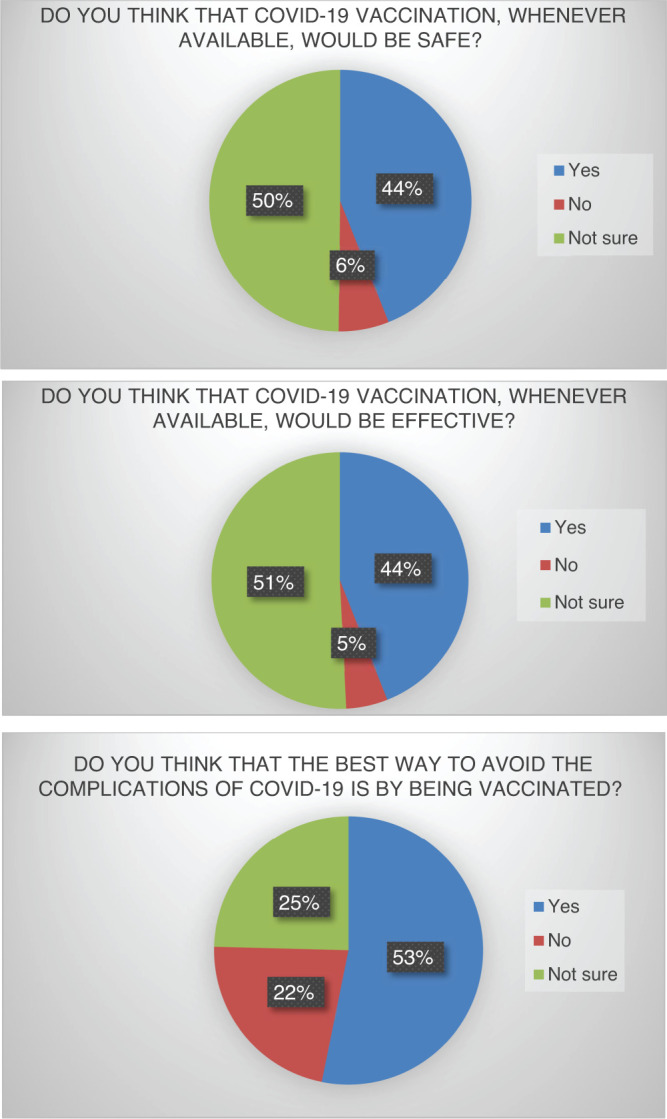
Pie diagrams showing the response of the participants regarding their beliefs (safety and effectiveness) towards COVID-19 vaccination. This figure appears in color at www.ajtmh.org.

**Figure 2.**
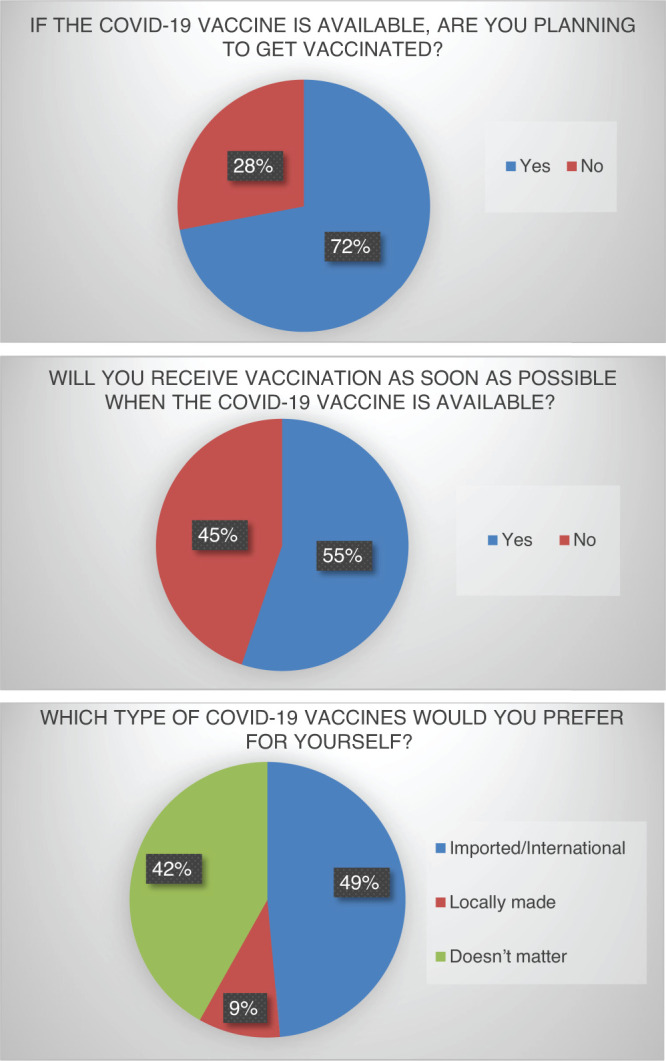
Pie diagrams showing the response of the participants regarding the acceptance of COVID-19 vaccination. This figure appears in color at www.ajtmh.org.

The beliefs of the participants regarding the COVID-19 vaccine (Figure 1) was accessed with three leading questions pertaining to the effectiveness, safety profile, and efficacy in preventing complications of the disease. About 782 (44%) of respondents believed the vaccine to be safe, 885 (50%) were not sure about it, and 111 (6%) considered it to be unsafe. Regarding the effectiveness, 783 (44%) considered it to be effective against the virus, 903 (51%) were unsure, and the remaining 92 (5%) believed the vaccine was ineffective. When asked whether the respondents believe the best way to avoid COVID-19 complications is by administering the vaccine, the highest proportion 946 (53%) answered in affirmative, 394 (22%) did not believe it, and 438 (25%) were not sure. Overall, 1,280 (72%) of participants planned to receive the COVID-19 vaccine, whereas 498 (28%) refused to get vaccinated. About 981 (55%) stated they would receive the vaccine as soon as it is available, whereas 797 (44%) would not. Upon asking their preference for different COVID-19 vaccines, 863 (49%) prioritized internationally made imported vaccines, 171 (9%) preferred locally made vaccines, while the type of vaccine did not matter to 744 (42%) of respondents (Figure 2).

### Barriers and potential recommendations that may improve COVID-19 vaccination.

**Figure 3.**
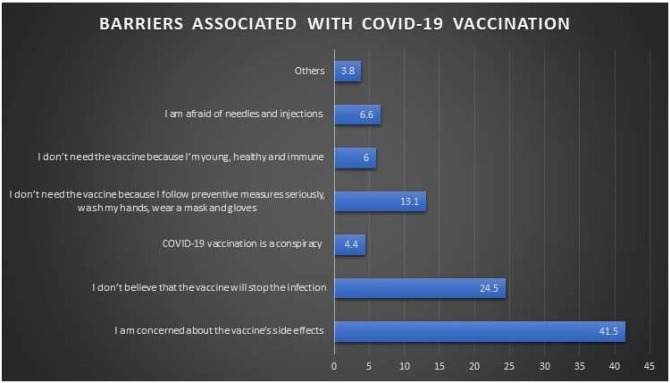
Response of the participants regarding the barriers associated with COVID-19 vaccination. This figure appears in color at www.ajtmh.org.

**Figure 4.**
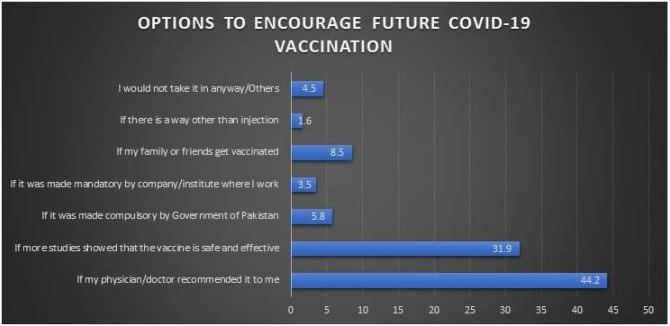
Response of the participants regarding the factors that can encourage public to get COVID-19 vaccination. This figure appears in color at www.ajtmh.org.

Regarding the barriers associated with COVID-19 vaccine acceptance (Figure 3), 738 (42%) were concerned about the vaccine’s side effects, 436 (25%) believed that the vaccine will not help against the virus, and only 79 (4%) labeled the vaccine as a conspiracy. Furthermore, 233 (13%) stated they do not need the vaccine because they follow all preventive measures seriously. The participants were asked for suggestions that might improve vaccination rates to which 785 (44%) agreed to receive it upon a physician recommendation, 568 (32%) required further validation from research studies to prove the efficacy and safety of the vaccine, 103 (6%) would receive the vaccine if made mandatory by the government, 62 (4%) would receive it if made mandatory by their affiliated organization/company, and 152 (9%) would get vaccinated upon the recommendation of their family/friends (Figure 4).

### Factors associated with the acceptance of COVID-19 vaccination.

On univariate regression models, male gender (odds ratio [OR]: 1.284 [95% CI: 1.029–1.602], *P* = 0.027), postgraduates (OR: 2.151 [95% CI: 1.181–3.917], *P* = 0.012), graduates (OR: 2.017 [95% CI: 1.312–3.100], *P* = 0.001), healthcare workers (OR: 2.398 [95%: 1.163–4.942], *P* = 0.018), residence of Sindh (OR: 2.128 [95% CI: 1.571–2.881], *P* < 0.001), residence of Punjab (OR: 1.621 [1.243–2.113], *P* < 0.001), and residence of Balochistan (OR: 1.661 [1.049–2.630], *P* = 0.030) were significantly associated with COVID-19 vaccination. Those who believed in the efficacy of polio vaccination (OR: 1.496 [95% CI: 1.055–2.121], *P* = 0.024), believed that the vaccine is safe (OR: 7.696 [95% CI: 5.735–10.327], *P* < 0.001), believed that vaccine is effective (OR: 3.565 [95% CI: 2.793–4.552], *P* < 0.001), believed vaccine is the only way to avoid COVID-19 complications (OR: 6.269 [95% CI: 4.693–8.373], *P* < 0.001), and preferred internationally made/imported vaccines (OR: 1.483 [95% CI: 1.189–1.850], *P* < 0.001) showed positive acceptance rates.

Lastly, factors including recommendation by a physician (OR: 10.847 [95% CI: 6.471–18.180], *P* < 0.001), further studies proving safety and efficacy of the vaccine (OR: 5.342 [95% CI: 3.191–8.944], *P* < 0.001), if made compulsory by government (OR: 6.638 [95% CI: 3.465–12.719], *P* < 0.001), if declared mandatory by affiliated institute/company (OR: 4.202 [95% CI: 2.072–8.522], *P* < 0.001), recommendation by a family member and/or friends (OR: 4.629 [95% CI: 2.517–8.334], *P* < 0.001), and way of administering other than injection (OR: 3.830 [95% CI: 1.558–9.418), *P* = 0.003) were significantly associated with vaccine acceptance (Table 2).

**Table 2:** Factors affecting COVID-19 vaccine acceptance among the participants

Study variables	If the COVID-19 vaccine is available, are you planning to get vaccinated?			
	Frequency		Univariate regression	
	Yes *N* (%) 1,280 (72)	No *N* (%) 498 (28)	OR (95% CI)	*P* value
Gender Male Female	470 (37) 810 (63)	155 (31) 343 (69)	1.284 (1.029–1.602) 1.000	0.027*
Age (years) 18–29 30–39 40–60	1,189 (93) 50 (4) 41 (3)	465 (93) 23 (5) 10 (2)	0.624 (0.310–1.255) 0.530 (0.227–1.240) 1.000	0.186 0.143
Education status Postgraduate level University graduate Higher secondary Primary/secondary school	82 (6) 609 (48) 528 (41) 61 (5)	25 (5) 198 (40) 235 (47) 40 (8)	2.151 (1.181–3.917) 2.017 (1.312–3.100) 1.473 (0.961–2.259) 1.000	0.012* 0.001* 0.076 –
Occupation Healthcare workers Students Govt/private employees Unemployed	99 (8) 1,036 (81) 94 (7) 51 (4)	17 (3) 422 (85) 38 (8) 21 (4)	2.398 (1.163–4.942) 1.011 (0.601–1.701) 1.019 (0.541–1.918) 1.000	0.018* 0.968 0.955 –
Marital status Married Single	114 (9) 1,166 (91)	42 (8) 456 (92)	1.062 (0.733–1.537) 1.000	0.752 –
Monthly income (Pakistani Rupee) < 20,000 21,000–50,000 51,000–100,000 101,000–200,000 > 200,000	131 (10) 210 (16) 342 (27) 313 (24) 284 (22)	64 (13) 78 (16) 131 (26) 132 (27) 93 (19)	0.863 (0.601–1.239) 1.135 (0.816–1.579) 1.101 (0.827–1.466) 1.000 1.288 (0.944–1.756)	0.425 0.451 0.510 – 0.110
Region Sindh Punjab Khyber Pakhtunkhwa Balochistan	383 (30) 594 (46) 216 (17) 87 (7)	110 (22) 224 (45) 132 (27) 32 (6)	2.128 (1.571–2.881) 1.621 (1.243–2.113) 1.000 1.661 (1.049–2.630)	< 0.001* < 0.001* – 0.030*
Any comorbidities Present Absent	61 (5) 1,219 (95)	24 (5) 474 (95)	1.012 (0.624–1.642) 1.000	0.962 **–**
Self-reported overall health Fair Good Excellent	164 (13) 714 (56) 402 (31)	71 (14) 265 (53) 162 (33)	0.931 (0.667–1.298) 1.086 (0.862–1.367) 1.000	0.673 0.484 **–**
What is your best guess as to whether you will get the coronavirus within the next 6 months? I think I will get a severe case of COVID-19 I think I will get a mild case of COVID-19 I don’t think I will get the coronavirus infection I have already had the coronavirus infection	41 (3) 445 (35) 572 (45) 222 (17)	21 (4) 142 (29) 252 (51) 83 (17)	0.623 (0.356–1.089) 1.000 0.724 (0.570–0.921) 0.853 (0.623–1.170)	0.097 – 0.324 0.008*
Do you believe in getting your children vaccinated against the polio virus? Yes No No response	177 (14) 285 (22) 818 (64)	46 (9) 134 (27) 318 (64)	1.496 (1.055–2.121) 0.827 (0.649–1.054) 1.000	0.024* 0.125 **–**
Do you think that COVID-19 vaccination, whenever available, would be safe? Yes No Not sure	721 (56) 23 (2) 536 (42)	61 (12) 88 (18) 349 (70)	7.696 (5.735–10.327) 0.170 (0.105–0.275) 1.000	< 0.001* < 0.001* –
Do you think that COVID-19 vaccination, whenever available, would be effective? Yes No Not sure	675 (53) 30 (2) 575 (45)	108 (22) 62 (12) 328 (66)	3.565 (2.793–4.552) 0.276 (0.175–0.436) 1.000	< 0.001* < 0.001* –
Do you think that the best way to avoid the complications of COVID-19 is by being vaccinated? Yes No Not sure	856 (67) 160 (13) 264 (21)	90 (18) 234 (47) 174 (35)	6.269 (4.693–8.373) 0.451 (0.341–0.595) 1.000	< 0.001* < 0.001* –
Will you receive vaccination as soon as possible when the COVID-19 vaccine is available? Yes No	940 (73) 340 (27)	41 (8) 457 (92)	30.816 (21.874–43.415) 1.000	< 0.001* –
Which type of COVID-19 vaccines would you prefer for yourself? Imported/International Locally made vaccine Doesn’t matter	661 (52) 107 (8) 512 (40)	202 (40) 64 (13) 232 (47)	1.483 (1.189–1.850) 0.758 (0.536–1.071) 1.000	< 0.001* 0.116 **–**
Barriers associated with COVID-19 vaccine acceptance I am concerned about the vaccine’s side effects I don’t believe that the vaccine will stop the infection COVID-19 vaccination is a conspiracy I don’t need the vaccine because I follow preventive measures seriously, wash my hands, wear a mask and gloves I don’t need the vaccine because I’m young, healthy, and immune I am afraid of needles and injections Others	607 (47) 258 (20) 52 (4) 132 (10) 72 (6) 98 (8) 61 (5)	131 (26) 178 (36) 27 (5) 101 (20) 35 (7) 20 (4) 6 (1)	0.456 (0.193–1.077) 0.143 (0.060–0.337) 0.189 (0.073–0.494) 0.129 (0.053–0.309) 0.202 (0.080–0.513) 0.482 (0.183–1.267) 1.000	0.073 < 0.001* 0.001* < 0.001* 0.001* 0.139 –
Options to encourage future COVID-19 vaccination If my physician/doctor recommended it to me If more studies showed that the vaccine is safe and effective If it was made compulsory by Government of Pakistan If it was made mandatory by company/institute where I work If my family or friends get vaccinated If there is a way other than injection I would not take it in anyway/Others	639 (50) 388 (30) 75 (6) 39 (3) 99 (8) 17 (1) 23 (2)	146 (29) 180 (36) 28 (6) 23 (5) 53 (11) 11 (2) 57 (11)	10.847 (6.471–18.180) 5.342 (3.191–8.944) 6.638 (3.465–12.719) 4.202 (2.072–8.522) 4.629 (2.517–8.334) 3.830 (1.558–9.418) 1.000	< 0.001* < 0.001* < 0.001* < 0.001* < 0.001* 0.003* –

* *P* ≤ 0.05 for all levels of statistical significance.

## DISCUSSION

In the 21st century, one of the most groundbreaking interventions in the healthcare system is the introduction of vaccination. This health security measure is facing numerous challenges worldwide, despite the known benefits of vaccination. The adoption of vaccination among the population is influenced by several variables, such as geography, time, social status, human behavior, and ethnicity.^24^^–^^26^ Public perception of the benefits and relative risks of vaccination have been reported to be a significant barrier to vaccine acceptance.^27^ Although a number of studies regarding COVID-19 vaccine acceptance are being undertaken worldwide, the number still remains limited at present. Our results show that vaccine acceptance was 72% in this study, which is in accordance with the results obtained from the US-based study that reported vaccine acceptance of the general population to be 80% while another study based in China reported vaccine acceptance to be 72.5% of the general population.^28^^,^^29^ Our study shows that the vaccine acceptance (72%) among participants was higher compared with some other studies, such as those conducted in South Africa (64%), Russia (54%), France (59%), Poland (56%), and Hungary (56%).^30^

Regarding willingness, the results showed that majority (55%) of the study population were willing to take the COVID-19 vaccine as soon as it is available, and this trend was noted to be similar to several other countries. Our study shows that 28% of the study population are reluctant in getting vaccinated upon its availability. IPSOS survey reported disapproval of the COVID-19 vaccine in 47% Russians, 41% French, 33% Italian, 30% Turkish, 33% Americans, 24% Canadians, and 15% English (Great Britain), 13% Indian, and 3% Chinese, once available.^31^ A survey conducted in Malaysia (a nation in Southeast Asia), with recorded COVID-19 cases of just over 4,000 and less than 100 deaths during the research period in April 2020, showed a higher (94.3%) intention to vaccinate, of which 48.2% stated a definite intention.^32^

The greatest obstacle that stands against the COVID-19 vaccine acceptance among the participants is the concerns regarding the adverse effects and efficacy of the vaccine, along with the lack of knowledge and belief in vaccines. This finding is consistent with studies of other vaccines.^33^ A poll conducted in the United States showed that if assurance regarding the safety of the COVID-19 vaccine was given, approximately 75% of the population would agree to get the vaccine.^34^ Some participants also believed that strict precautionary measures (regular handwashing and the usage of gloves and masks) were enough to replace the need to get the vaccine entirely.

Based on the overall response, the following options would encourage future COVID-19 vaccinations and acceptance, that is, if more physicians started to recommend the vaccines to their patients, and if more vaccine-related studies showed safety and effectiveness. Additionally, information regarding vaccine safety should be disseminated in a straightforward manner, so that it can be interpreted by individuals at all levels of education to improve vaccine acceptance by the public. Moreover, there is evidence that people whose physicians recommend vaccines are more likely to get vaccinated compared with ones who are not recommended.^35^ It has been argued that physicians are more suited and qualified to address concerns, side effects, and misinformation regarding the vaccine, and to also convey the magnitude of COVID-19 infection in a tailored manner for each patient.^36^ However, owing to the time constraints at primary healthcare encounters, and the potential need for staff training to address vaccine-related concerns, healthcare providers should explore an alternative approach in which vaccine counselors use motivational interviewing to approach vaccine-hesitant individuals. This approach has been successful at increasing rates of infant vaccine coverage and adolescent human papillomavirus vaccination.^37^

This cross-sectional survey was conducted using a validated, self-administered electronic questionnaire that was distributed to the general public of Pakistan via different online platforms, and therefore has certain limitations. Firstly, the lack of Internet availability and access to social media platforms may have had an impact on the survey population due to which a bias in reporting results should be considered. Secondly, the difference in public perceptions that may be associated with sociodemographic factors were not addressed in the study. Lastly, the sample size may not be large enough to represent the entire Pakistani population, so the results should be carefully generalized.

Nevertheless, the major findings of this study can be used in planning potential vaccination campaigns. The study identifies concerns among the participants regarding the safety and efficacy of COVID-19 vaccines, and hence provides a valuable important perspective for possible interventional educational programs to enhance vaccination rates. This study also serves an important baseline to conduct further studies in a larger population to obtain an insight on the behavior, and public perception of upcoming COVID-19 vaccines. Lastly to the best our knowledge, this is the first study conducted to determine the COVID-19 vaccine acceptance rates on a large population of 1,778 citizens, and represents participants from all four provinces of Pakistan.

## CONCLUSION

Reasons for vaccine hesitancy should further be evaluated by the government to ensure more acceptance of the COVID-19 vaccine by the public. Herd immunity can only be achieved if the beliefs regarding vaccine safety and concerns are addressed. Positive and accurate information regarding the COVID-19 vaccine needs to be disseminated through physicians, and the social media, to the general public.
